# Practical Medicine

**Published:** 1879-08

**Authors:** 


### Summary.

*Collaborators:*

DR. H. GRADLE, DR. L. W. CASE, DR. R. PARK,

DR. R. TILLEY.

### PRACTICAL MEDICINE.

LARYNGEAL VERTIGO. Report of Clinic of M. Charcot. (*Le Progrès Médical*, April 16, 1879).

The above phrase was used by J. R. Gasquet in the *Practitioner* of August, 1878, but its corresponding French term, *Vertige Laryngé* was used by Dr. Charcot in a communication to the Society of Biology, Nov. 19, 1876. The following case is given as a specimen of several in virtue of which the name was suggested:

M. G., age 45; first attacked on the 20th July, 1878, with an ordinary bronchitic affection, which rapidly yielded to treatment, but there remained in the region of the larynx an almost constant tickling and burning sensation. This sensation increased, occasionally, all at once, and was followed by violent paroxysms of coughing. In the month of August M. G. was suddenly awakened out of sleep by one of these paroxysms. He got up, lost consciousness almost immediately, and a few minutes later recovered and found himself lying upon the floor. From this time these crises frequently returned, sometimes three or four times a day. Consciousness was always lost; sometimes he fell in the street, coming to himself in a few minutes without any derangement of ideas, but at the same time without any memory of what took place during the attack, remembering only the tickling sensation of the throat and the immediate appearance of vertigo.

According to the testimony of attendants, his face became congested and somewhat purple. Occasionally convulsive movements appeared in the face and limbs; no initial cry, no involuntary discharge of urine. He never bit his tongue and never experienced nausea or vomiting at the time of recovery. Laryngoscopic examination revealed no lesion.

The trouble disappeared in three weeks on the use of flying blisters to the region of the larynx, and heavy doses of bromide of potassium.

An observation, referred to by Dr. Charcot, recorded by Dr. Sommerbrodt (*Berliner Klin-Wochenschrift*, 25th Sep. 1876), informs us of a man, age 54 years, for some time the subject of attacks of apparent epilepsy with special laryngeal symptoms. A tumor of the larynx having been recognized and removed, the apparent epileptic attacks ceased. This case is quoted to show that such attacks may be brought on by an irritation of the larynx. In the other cases given no lesion of the larynx was observed.

Dr. Charcot recognizes two degrees in these attacks corresponding to the *haut mal* and *petit mal* of epilepsy. In the former case, complete loss of consciousness takes place, though it lasts but a few seconds, scarcely minutes; certain convulsive movements of the face were well marked. In the latter class the exciting cause fails to produce complete loss of consciousness and the other phenomena associated with the greater disturbance.

Its differential diagnosis from the true epileptic attack and from similar consequences associated with affections of the labyrinth is: it is always associated with a tickling and burning sensation or other irritation in the region of the larynx or upper part of the trachea. The loss of consciousness is of short duration; there is no stupid expression on the countenance at the close of the attack; no nausea or vomiting; no biting of the tongue; no involuntary discharge of urine, and no excessive sensibility of the organ of hearing preceding the attack.

